# Effects of Highly Pathogenic Porcine Reproductive and Respiratory Syndrome Virus Infection on the Surface Glycoprofiling of Porcine Pulmonary Microvascular Endothelial Cells

**DOI:** 10.3390/v14112569

**Published:** 2022-11-20

**Authors:** Xiaoxiao Song, Yanmei Wu, Xianping Wu, Ge Hu, Tao Zhang

**Affiliations:** Beijing Key Laboratory of Traditional Chinese Veterinary Medicine, Animal Science and Technology College, Beijing University of Agriculture, Beijing 102206, China

**Keywords:** porcine reproductive and respiratory syndrome virus, pulmonary microvascular endothelial cells, lectin microarray, poly-N-acetyllactosamine, complex N-glycan

## Abstract

Previously, our study has demonstrated that porcine pulmonary microvascular endothelial cells (PPMVECs) were susceptible to highly pathogenic porcine reproductive and respiratory syndrome virus (HP-PRRSV) and produced a significant non-specific immune response to it. The significance of microvascular endothelial glycocalyx is increasingly attracting attention, and its rich carbohydrate components are not only important signaling molecules, but also remarkably influence the signaling of most proteins. Comprehending changes in the carbohydrate chains contributes to understanding cell functions. This study aimed to reveal the effects of HP-PRRSV infection on the surface carbohydrate chains of PPMVECs. PPMVECs were isolated and cultured in vitro and infected with HP-PRRSV HN and JXA1 strains. Scanning electron microscopy analysis indicated that at 48 h post-infection, some broken holes were in their cell membranes, and that the surface fibrous glycocalyx was obviously reduced or even disappeared. Lectin microarray analysis indicated that the fluorescence intensities of 8 and 7 lectin sites were significantly changed by the HP-PRRSV HN and JXA1 strains, respectively, among which there were 6 common lectin sites. The up-regulation of common lectins (RCA-I, LEL, and STL) and the down-regulation of common lectins (LCA, DSA, and PHA-E) were confirmed by lectin fluorescence staining and lectin flow cytometry, respectively. Together, the results show that the HP-PRRSV infection can induce the glycocalyx disruption of PPMVECs and their surface glycoprofiling changes, and that the poly-N-acetyllactosamine and complex N-glycan are the main up-regulated and down-regulated carbohydrate chains, respectively. Our findings may provide insights into revealing the pathogenesis of HP-PRRSV from the perspective of glycobiology.

## 1. Introduction

Porcine reproductive and respiratory syndrome virus (PRRSV) has been widespread around the world and has seriously jeopardized the pig industry for decades, mainly causing severe reproductive disorders in sows and respiratory symptoms in piglets [1]. However, its pathogenesis has not been fully clarified so far, causing its prevention and control to remain a great challenge. Particularly, the highly pathogenic PRRSV (HP-PRRSV) emerged in 2006 in China and spread to become the dominant strain in most Southeast Asia countries, which caused more severe lesions and higher mortality in infected pigs [2]. Thus, greater pressure has been put on the prevention and treatment of HP-PRRSV, and huge economic losses have been caused to the pig industry. To eradicate the threat, it is necessary to comprehensively study its pathogenesis. The current research has mainly focused on alveolar macrophages as the main target cell of HP-PRRSV [3]. Although many significant achievements have been achieved, we believe that the complex pathological changes and pathogenesis cannot be fully explained only from alveolar macrophages. Other kinds of cells possess critical roles in physiological processes and pathological changes should also be paid attention to.

Microvascular endothelium has diverse biological functions, which are not only the physical barrier of blood, but also the key pivot for a variety of basic physiological processes and pathological changes in the bodies. In recent years, its crucial role in viral infections has received increasing attention, and has been demonstrated in the studies of various respiratory viruses, such as SARS-CoV-2 [4], influenza virus [5], and swine influenza virus [6]. The endothelial glycocalyx is a carbohydrate-rich villous structure lining the luminal surface of blood vessels, which is involved in most biological functions of microvascular endothelial cells (MVECs) [7]. Glycocalyx damage due to viral infection is the key reason for microvascular endothelial dysfunction [8,9]. Its surface carbohydrate components not only support and protect the cells [10], but also affect cellular protein molecule functions through glycosylation. Thus, they can influence cellular behavior and gene expression [11] and participate in mediating viral invasion [12]. Therefore, studying the viral infection-induced alteration of host cell surface carbohydrate chains will contribute to fully clarifying the pathogenesis. Our previous studies demonstrated that PPMVECs were susceptible to HP-PRRSV [13] and produced a significant non-specific immune response to it [14], which shows that PPMVECs are also crucial target cells of HP-PRRSV, and the effects of this virus infection on their surface carbohydrate chains deserve further study.

Lectins can specifically recognize carbohydrate structures, and are generally used to detect carbohydrate chains. Lectin microarrays are an important tool for glycomics research [15]. The specificity and affinity of lectins and carbohydrate chains are lower than those of antigens and antibodies, and the kinds of available lectins are less. However, lectin microarray technology is rapid and high-throughput and suitable for the detection of surface carbohydrate chains in whole cells and even living cells, so it shows great potential for the cell surface glycan profiling analysis. Furthermore, the differentially expressed carbohydrate chains obtained from lectin microarrays need to be further validated by other tests, such as lectin fluorescence and lectin flow cytometry, and the complete and precise structure of carbohydrate chains can be analyzed by other techniques, such as mass spectrometry and liquid chromatography.

To understand the effects of HP-PRRSV infection on the surface carbohydrate chains of PPMVECs, they were infected with HP-PRRSV HN and JXA1 strains in this study. Then, their surface ultrastructure was observed by scanning electron microscopy, their surface carbohydrate chains glycoprofiling was analyzed by lectin microarrays, and the differentially expressed carbohydrate chains were confirmed by lectin fluorescence staining and lectin flow cytometry, respectively. Our findings reveal the responses of PPMVECs to HP-PRRSV infection from the perspective of glycobiology and provide an experimental basis for further elucidating the pathogenesis of HP-PRRSV.

## 2. Materials and Methods

### 2.1. Viruses

The HP-PRRSV strain JXA1 and HN used in this study were gifted from Dr. Zhanzhong Zhao of the Chinese Academy of Agricultural Sciences, and propagated and titrated on Marc-145 cells in Dulbecco’s Modified Eagle’s Medium (DMEM, Gibco, H2387, Carlsbad, CA, USA) supplemented with 10% fetal bovine serum (FBS, Gibco, 10099-141, Carlsbad, CA, USA) and 2 mM L-glutamine at 37 °C in an incubator humidified with 5% CO_2_. At 72 h after infection, the centrifuged virus suspension was collected through multiple freeze-thaw cycles, and the viral titers of 4.4 logs TCID_50_ and 6.3 logs TCID_50_ were detected in the infected Marc-145 cells, respectively. The normal Marc-145 cell lysate was collected in the same way as the control medium, which was stored at −80 °C and then used to infect PPMVECs.

### 2.2. Cell Culture and Treatments

PPMVECs were separated from the peripheral lung tissues of 10-day-old SPF piglets, which were purchased from the Beijing Center for Specific Pathogen-free Swine Breeding and Management and tested negative for HP-PRRSV. Cells were purified using the immunomagnetic beads coated with CD31 antibodies as previously described with minor modifications [13,16].

In brief, the lung tissues were sterilely removed from the piglets and the pleura were torn away. The minced tissues were digested in 0.2% collagenase type II solution for about 60 min at 37 °C, the filtrate through the 100-µm cell strainer was centrifuged. The cell pellet was resuspended in DMEM containing 20% FBS, 2 mM L-glutamine, 100 U/mL penicillin, and 100 U/mL streptomycin, and plated in 6-well plates. After about 2 h incubation, the non-adherent cells were rinsed off and the complete medium was replaced, which was changed every 3 days until the cells grew to confluence. Meanwhile, the contaminating fibroblasts were removed by mechanically scraping in the light of their elongated morphology and multilayer growth. The confluent primary cell culture was detached and incubated with anti-CD31 antibodies (Proteintech, 11265-1-AP, Rosemont, IL, USA) for about 30 min at room temperature. After washing, cells conjugated with CD31 antibodies were incubated with pre-washed beads (Thermo Fisher, 11531D, Waltham, MA, USA) for 30 min. The CD31^+^ cells were separated using KingFisher 96 automatic magnetic separation system (Thermo Fisher, Vantaa, Finland), and then the beads were released using the DNase I solution. The released cells were plated and cultured as above.

All experiments were performed with the purified MVECs in triplicate at passage 3–5. Cells were grown on coverslips in 24-well plates for 48 h and fixed with 4% paraformaldehyde (Leagene, DF0135, Beijing, China) for immunofluorescence staining. Cells were grown on coverslips to subconfluence and incubated in the maintenance medium containing 2% FBS overnight, and then infected with HP-PRRSV. After 48 h incubation, the cells were fixed with 2.5% glutaraldehyde (Leagene, DF0151, Beijing, China) and 4% paraformaldehyde mixed in equal proportions for lectin fluorescent staining, or fixed with 2.5% glutaraldehyde for scanning electron microscope. Cells were grown in dishes to subconfluence and incubated in the maintenance medium overnight, and then infected with HP-PRRSV. After 48 h incubation, the cells were scraped and centrifuged into pellets for lectin microarray analysis or digested with 0.25% trypsin solution and centrifuged for flow cytometry assays.

### 2.3. Immunofluorescent Staining

Indirect immunofluorescence staining was performed to detect the expression of factor VIII (FVIII). In brief, the fixed cells were washed and blocked with 0.01M PBS containing 3% BSA for 30 min, and then incubated with anti-FVIII antibodies overnight at 4 °C (Bioss, BS-2974R, Beijing, China). In the negative control group, the antibody was replaced with PBS. After washing, FITC-conjugated goat anti-rabbit IgG (Proteintech, SA00003-2, Rosemont, IL, USA) was added and incubated for 2 h at room temperature in the dark. Cell nuclei were counterstained with DAPI (Beyotime, C-1006, Beijing, China), and cells were washed and mounted in glycerol. The micrographs were obtained with a fluorescence microscope (IX71, Olympus, Tokyo, Japan) at 490 and 350 nm, respectively, and then the single-channel images were merged.

### 2.4. Scanning Electron Microscope

The fixed cells were washed and further fixed with 1% osmic acid for 1.5 h at 4 °C. After washing, gradient dehydration was performed with ethanol of 50%, 70%, 80%, 90%, and 100%, which was replaced with 100% tert-butyl alcohol at −20 °C for 30 min. The cell samples were dried for 2 h with a critical dryer (Emitech k-850, Ashford, Kent, UK) and then stuck on the sample table with conductive adhesive. Cells were sprayed with an ion-sputtering instrument (Hitachi e-1010, Tokyo, Japan) and observed under the scanning electron microscope (Tescan 5136, Brno, Czech Republic) at the accelerating voltage of 10~15 kv.

### 2.5. Lectin Microarrays

Lectin microarray analysis was carried out by Creative Biochip (Nanjing, China). The lectin microarray contained 26 lectins and each lectin had triplicate spotting blocks. The cell pellets were washed with TBST and dissociated with the dispase solution, and labeled with Cy3 (GE Healthcare, Buckinghamshire, UK). After incubation on a shaker in the dark for 1 h, the cells were dialyzed three times by Slide-A-Lyzer™ (Thermo Fisher, 88401, Waltham, MA, USA). The internal between the first two dialyses was 1 h, and the last one was maintained overnight at 4 °C. The cells were resuspended in 0.01 M PBS containing 0.1% tween-20 and 1% BSA at the concentration of 1 × 10^6^ cells/mL. The 100 μL blocking buffer (0.01 M PBS containing 1% BSA) was added to the microarrays and incubated for 30 min on a shaker. After removing the blocking buffer, the 100 μL sample solution was added and incubated for 1 h on a shaker in the dark. After washing, the microarrays were incubated with TBST for 10 min and with deionized water for 2 min. The microarrays were dried by centrifugation and then scanned using the LuxScan 10K-A microarray chip scanner (CapitalBio, Beijing, China) with 5 μm resolution.

The acquired images were analyzed for Cy3 detection at 532 nm by Genepix 3.0 software (Axon Instruments, Inc, San Francisco, CA, USA). The signal intensity values were calculated from the total intensities by subtracting the background intensities, which would be considered invalid values and excluded in subsequent analysis when their values were negative. The ratios of each lectin to the sum of 26 lectins were calculated to normalize the data. The differentially expressed lectins were defined as the fold change >1.5 or <0.67 and *p* < 0.05, and the hierarchical clustering analysis was performed with the online software (http: //www.bioinformatics.com.cn/, accessed on 17 November 2022).

### 2.6. Lectin Fluorescent Staining

The fixed PPMVECs were washed and blocked with 10% albumen solution for 30 min. After washing, cells were incubated with FITC-conjugated lectins overnight at 4 °C. LCA (FL-1041), STL (FL-1161), RCA-I (FL-1081), LEL (FL-1175), PHA-E (FL-1211), and DSA (FL-1181) were purchased from Vector Laboratories Inc (Burlingame, CA, USA) and used at the concentration of 6 μg/mL. For their hapten sugar blocking controls, the lectins were preincubated for 1 h with 0.2 M methyl-alpha-D-mannopyranoside (Alfa Aesar, A11533, Ward Hill, MA, USA), N-acetyl-D-glucosamine (Sigma Aldrich, A8625, Saint Louis, MO, USA), D-(z)-galactose (Sigma Aldrich, G0625), N-acetyl-D-glucosamine, D-(z)-galactose and N-acetyl-D-glucosamine, respectively. The cell nuclei were counterstained with DAPI, and cells were washed and mounted in glycerol. The micrographs were obtained with the Olympus IX71 microscope at 490 and 350 nm, respectively, and then the single-channel images were merged.

### 2.7. Lectin Flow Cytometry

Lectin flow cytometry was performed as previously described [17]. The single-cell suspension of PPMVECs in PBS was prepared and incubated with FITC-conjugated lectins at the concentration of 6 μg/mL at 4 °C in the dark for 1 h. The lectin solution was replaced with PBS as a negative control. After washing 3 times, the cells were resuspended with 500 μL PBS and detected by flow cytometry (BD FACSCalibur, Franklin, NJ, USA).

### 2.8. Statistical Analysis

Statistical analysis was performed by GraphPad Prism 8.0. All experiments were performed with at least 3 biological replicates. Data were expressed as the means ± standard deviations, and *p* values were calculated using one-way ANOVA or Student’s *t*-test between two groups. Statistical significance was declared at *p* < 0.05.

## 3. Results

### 3.1. Characteristics of PPMVECs

**Figure 1:**
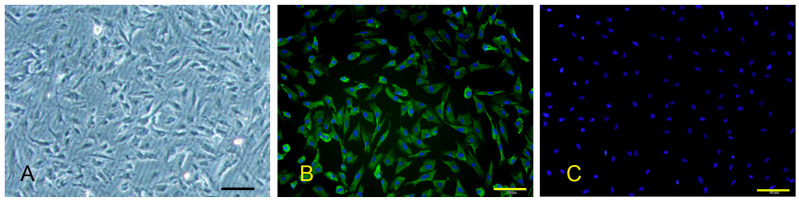
Microscopic morphology and FVIII immunofluorescence staining of PPMVECs. Bar = 50 μm. (**A**) Microscopic morphology. (**B**) Positive staining for FVIII. (**C**) Negative control of FVIII immunofluorescence staining.

The purified PPMVECs had a polygonal microscopic morphology, and grew into a confluent monolayer over about 5-day culture (Figure 1A). They maintained a good growth status until passage 6, and aged rapidly after passage 8. Immunofluorescence staining showed that they were positive for FVIII, and the positive rate was about 99% (Figure 1B).

### 3.2. Ultrastructural Observation of HP-PRRSV-Infected PPMVECs

**Figure 2:**
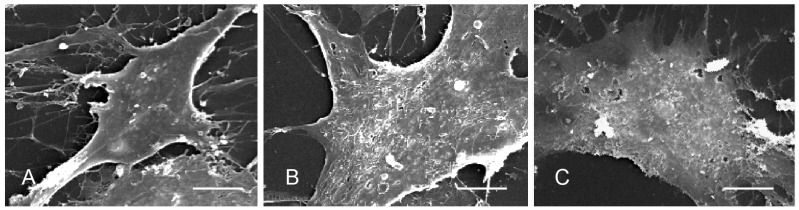
Ultrastructure analysis of HP-PRRSV-infected PPMVECs by scanning electron microscopy. (Bar = 4 μm). (**A**) Normal control group. (**B**) HP-PRRSV HN-infected group. (**C**) HP-PRRSV JXA1-infected group.

To understand the general effect of HP-PRRSV infection on the glycocalyx of PPMVECs, the surface ultrastructure was observed by scanning electron microscopy. The results showed that normal cells had a compact cell body, intact cell membrane, several cellular processes, and lots of filamentous structures on the cell surface (Figure 2A). After 48 h of infection with HP-PRRSV HN and JXA1 strains, the cell size swelled, and the integrity of the cell membrane was impaired. Many holes were on the membrane, and the surface filamentous glycocalyx was obviously reduced or even disappeared (Figure 2B,C). The changes in cell ultrastructure indicated that the cell membrane and surface glycocalyx structure of PPMVECs were significantly damaged by HP-PRRSV infection.

### 3.3. Changes in Surface Glycoprofiling of HP-PRRSV-Infected PPMVECs

**Figure 3:**
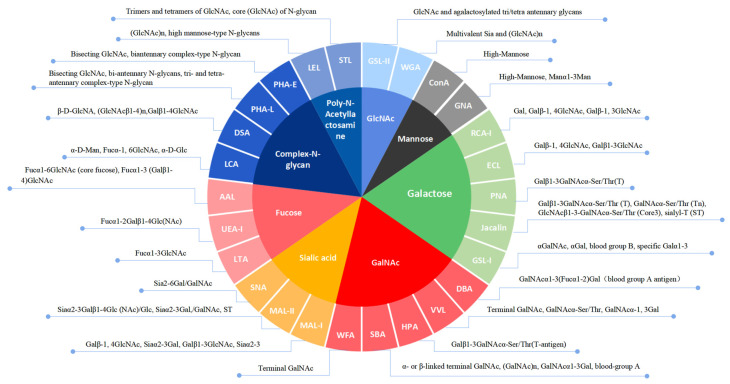
Lectin information in the lectin microarray.

**Figure 4:**
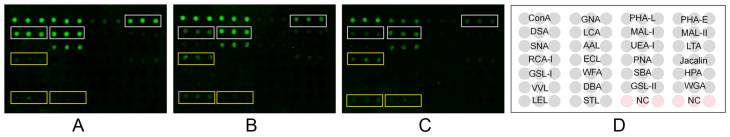
Scans of the lectin microarray. (**A**) Normal control group. (**B**) HP-PRRSV HN-infected group. (**C**) HP-PRRSV JXA1-infected group. (**D**) The layout of the lectin microarray (see Table S1 for full names). Each lectin was spotted in triplicate per block. Yellow frames and white frames marked indicated the lectin sites significantly increased and decreased compared to the normal control group, respectively. The negative controls showed no positive signal. NC: negative control.

To grasp the surface glycoprofiling changes in HP-PRRSV-infected PPMVECs, the lectin microarray containing 26 lectins, which bind 8 kinds of carbohydrate chain residues (Figure 3), was used, and its layout was shown in Figure 4D. The result showed that most fluorescence areas were uniform dots, and that the fluorescence intensities of triplicate sites had good repeatability. The background fluorescence signal was low and had no significant effects on the data. No fluorescence signal was detected in the negative controls. The fluorescence signal intensities of 26 lectins were significantly different in normal PPMVECs. The strong fluorescence signals were obtained at 6 sites of Con A, LCA, PHA-E, GNA, DSA, and AAL, the moderate signals at 5 sites of PHA-L, Jacalin, LEL, RCA-I, and MAL-II, and the weak signals at 6 sites of MAL-I, SNA, UEA-I, STL, VVL, and GSL-I. No fluorescence signals were captured at the other nine lectin sites. The result indicated that the cell surface carbohydrate chains were abundant in complex-N-glycan, mannose, galactose, sialic acid, and fucose. After infection with HP-PRRSV HN and JXA1 strains, the general fluorescence intensity pattern was approximately the same as that of the normal cells, while the fluorescence intensities of several lectin sites showed significant changes.

**Figure 5:**
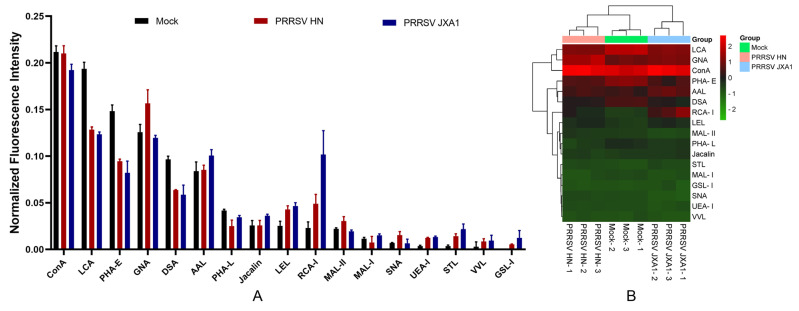
Fluorescence intensity analysis of the lectin microarray. (**A**) Normalized values of fluorescence signal intensities at positive lectin sites. (**B**) Heat map and hierarchical clustering of the positive lectins in PPMVECs. Samples are listed in columns and lectins in rows.

**Figure 6:**
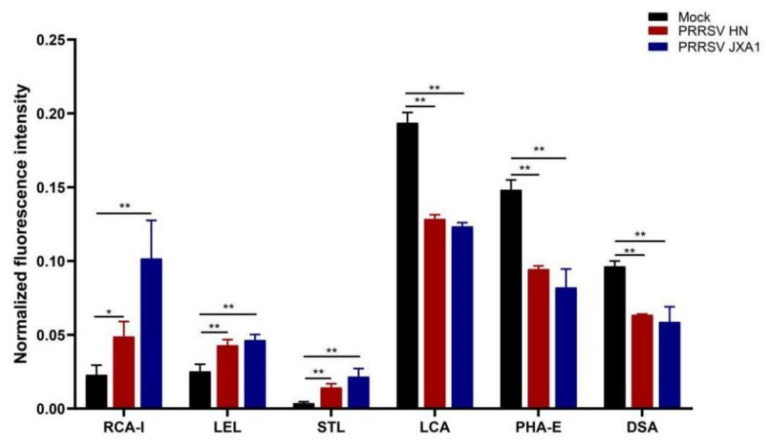
Differentially expressed lectin sites in both HP-PRRSV strain-infected PPMVECs. Statistical analysis was performed by Student’s *t*-test. * *p* < 0.05; ** *p* < 0.01.

The statistical analysis showed that the fluorescence intensities were significantly changed by the HP-PRRSV infection at some lectin sites (Figure 5). The HP-PRRSV JXA1 strain induced four up-regulated lectins and three down-regulated lectins, and the HP-PRRSV HN strain induced four up-regulated lectins and four down-regulated lectins (Table 1). Interestingly, RCA-I, LEL, and STL were increased in both HP-PRRSV strains, and LCA, PHA-E, and DSA declined in both HP-PRRSV strains (Figure 6). Lectin microarray analysis showed that HP-PRRSV infection induced significant surface glycoprofiling changes in PPMVECs, and the up-regulated carbohydrate chains mainly included poly-N-acetyllactosamine, galactose, sialic acid, and fucose, and the down-regulated ones mainly included complex N-glycans.

### 3.4. Lectin Fluorescence Staining of PPMVECs

**Figure 7:**
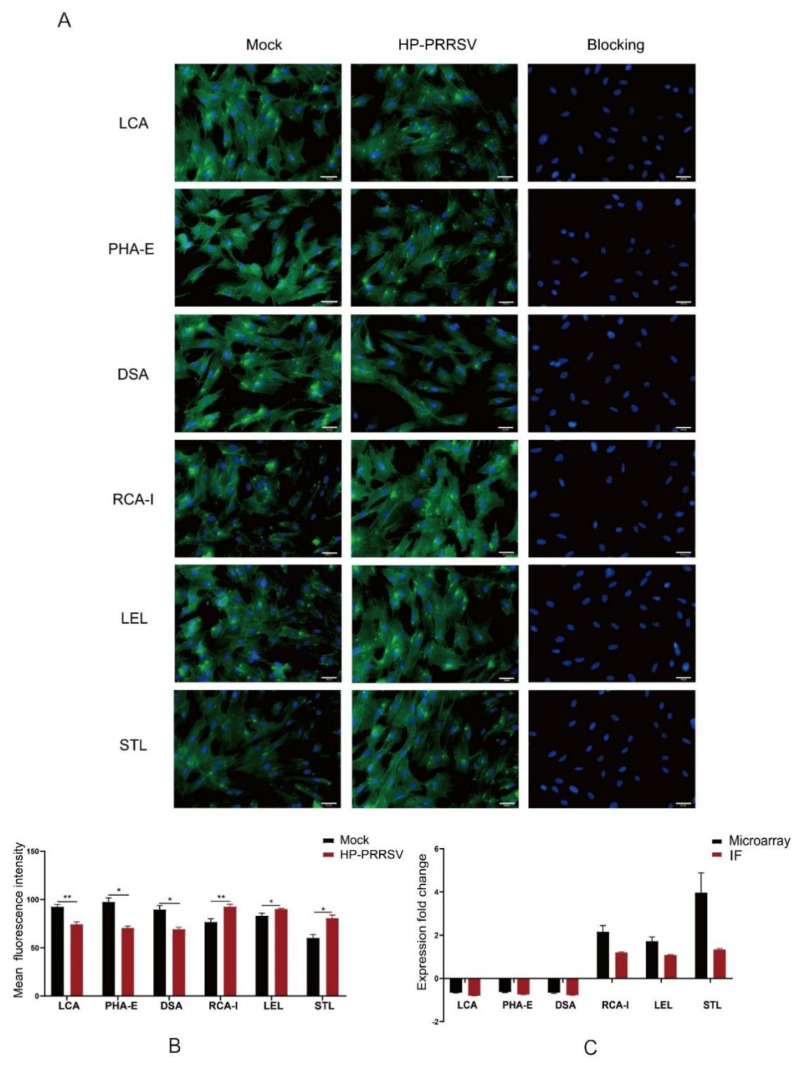
Lectin fluorescence staining of HP-PRRSV HN infected PPMVECs. Bar = 20 μm. (**A**) Photographs of lectin fluorescence staining. (**B**) Mean intensities of lectin fluorescence staining. (**C**) Comparison of the fold change in _-_fluorescence intensities between lectin microarrays and lectin fluorescence staining (IF). Statistical analysis was performed by Student’s *t*-test. * *p* < 0.05; ** *p* < 0.01.

To validate the results of the lectin microarrays, the lectin fluorescence staining of six lectins differentially changed in both HP-PRRSV strains was performed in HP-PRRSV HN-infected PPMVECs. The positive staining was observed in all six lectins, and in normal cells, LCA and PHA-E displayed strong fluorescence, DSA moderate fluorescence, RCA-I, LEL, and STL weak fluorescence. At 48 h after infection, the fluorescence intensities of RCA-I, LEL, and STL significantly increased, and those of LCA, PHA-E, and DSA significantly decreased (Figure 7). The results showed that the fluorescence intensity pattern of six lectins and the effects of HP-PRRSV HN strain on their intensities were consistent with the results in lectin microarrays.

### 3.5. Lectin Flow Cytometry of PPMVECs

**Figure 8:**
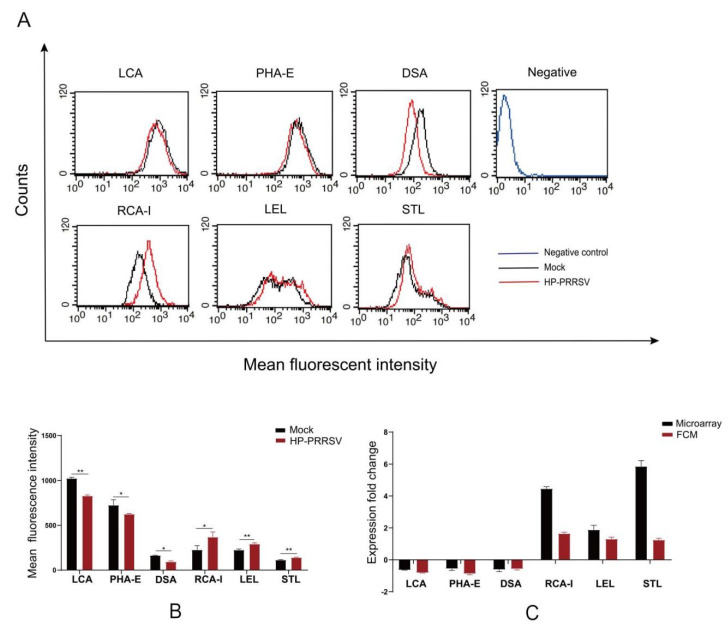
Lectin flow cytometry of HP-PRRSV JXA1-infected PPMVECs. (**A**) Flow cytometry histograms. (**B**) Mean fluorescence intensities. (**C**) Comparison of the fold changes in fluorescence intensities between lectin microarrays and lectin flow cytometry (FCM). Statistical analysis was performed by Student’s *t*-test. * *p* < 0.05; ** *p* < 0.01.

To further verify the results of the lectin microarray, lectin flow cytometry of six common lectins differentially changed in both HP-PRRSV strains was performed in HP-PRRSV JXA1-infected PPMVECs. No obvious fluorescence signal was detected in the negative control. Compared with the normal group, the fluorescence intensities of LCA, PHA-E, and DSA decreased significantly, and those of RCA-I, LEL, and STL increased significantly (Figure 8). The results confirmed the effects of HP-PPRSV on the surface carbohydrate chains of PPMVECs.

## 4. Discussion

Glycobiology is an indispensable part of the life sciences, and many problems in the life sciences are not only at the level of proteins and nucleic acids, but also closely related to the carbohydrate chains. The carbohydrate chains on the surface of MVECs are the main component of their glycocalyx, directly involved in intercellular communication and molecular recognition, which are crucial to the regulation of protein molecular functions [18]. Research in the effects of viral infection on the cell surface carbohydrate chains can contribute to a comprehensive understanding of the host cell responses and viral pathogenesis. Recent studies on SARS-CoV-2 have made the significance of glycocalyx carbohydrate chains emerge once again [19], and the unknown roles of carbohydrate chains may account for many unsolved diseases including COVID-19. In this study, the surface ultrastructure and carbohydrate chain expression of PPMVECs were investigated, and it was found that the HP-PRRSV infection induced the disruption of glycocalyx and alteration of surface glycofiling. The results suggest that the dysfunction of PPMVECs would occur and the potential roles of their glycocalyx carbohydrate chains in the pathogenesis of HP-PRRSV deserve further studies.

Glycocalyx plays a crucial role in the endothelial barrier, filtration, and communication between the endothelium and inflammatory and immune cell in the blood. Its disruption indicates impaired endothelial functions. The scanning electron microscopy observations showed abundant filamentous glycocalyx structures on the membrane surface of normal PPMVECs and the broken holes and reduced filamentous structures in HP-PRRSV-infected PPMVECs, which suggested the glycocalyx of PPMVECs was damaged. The effect of HP-PRRSV infection was similar to that of LPS in Yuan’s previous study [20]. Our results suggest that protecting the glycocalyx of PPMVECs to maintain their normal function should be a key point in the prevention and treatment of HP-PRRSV. Given that carbohydrate chains are important components of the glycocalyx and that the disruption of glycocalyx would result in changes in carbohydrate chains, the surface glycoprofiling of HP-PRRSV-infected PPMVECs was further analyzed. 

In the lectin microarray analysis, although more lectin sites were changed by infection of HP-PRRSV HN strain than HP-PRRSV JXA1 strain, both virus strains shared most differentially expressed lectin sites, namely, the up-regulated lectins mainly binding poly-N-acetyllactosamine and galactose and the down-regulated lectins mainly binding complex N-glycans. To know the binding patterns of lectins and confirm the changes in their fluorescence intensities, the differentially expressed lectins were validated by lectin fluorescence staining and lectin flow cytometry, respectively. The lectin fluorescence staining results showed the same fluorescence intensity patterns with lectin microarrays. For the lectin flow cytometry assay, we labeled the living cells with fluorescein-conjugated lectins to better preserve the carbohydrate chains. The different methods were in agreement with the lectin fluorescence intensity changes. The results from lectin microarrays were confirmed by both validations.

Although the biological functions of complex N-glycans and poly-N-acetyllactosamine are still poorly understood, an increasing number of studies have shown that they are involved in molecular recognition and signal transduction, and play important roles in immune signal transduction, pathogen binding, cell membrane stability, tumor metastasis, and cell proliferation and differentiation [21]. Poly-N-acetyllactosamine is a skeleton modified by a variety of terminal carbohydrate chains, and the formed glycan structure is the major component of matrix proteins such as fibronectin and many transmembrane glycoproteins on the cell surface [22]. It was widely documented that poly-N-acetyllactosamine was expressed in vascular endothelial cells [23] and involved in immune cell trafficking and tumor metastasis by binding specifically to selectins or affecting the functions of inflammatory molecules such as integrins and chemokine receptors through post-translational glycosylation modifications [24]. Complex N-glycans modify almost all cellular surface and secretory proteins in humans, showing an irreplaceable role in protein modification [25]. The reduction in complex N-glycans means the lowering of N-glycan branching and its size, which would minimize their spatial structure. Then, the binding of endothelial ICAM-1 binding to CD11b would be facilitated and the adhesion of monocytes to vascular endothelial cells would be increased [26]. In contrast, the anti-inflammatory laminar shear stress can promote the production of complex N-glycans [27]. Accordingly, both the decrease in complex N-glycans and the increase in poly-N-acetyllactosamine contribute to inflammation, which suggests that the role of protein glycosylation in PRRSV-induced inflammatory lung injury in piglets deserves further studies.

In addition, the fluorescence signals of lectins specifically binding sialic acid and fucose were significantly up-regulated when HP-PRRSV HN and JXA1 strains were infected, respectively. Sialic acid is widely expressed on various cell surfaces. It not only is one of the receptors mediating the virus invasion of cells, such as PRRSV [28], but also involved in immune regulation such as leukocyte trafficking and immune cell activation [29]. The fucosylated glycans are generally considered key to intercellular interactions and signaling processes, and are particularly well studied in cell adhesion [30]. It was reported that fucosylation of endothelial mucins regulated IL-1β-induced monocyte-endothelial adhesion, which promoted the initiation and development of atherosclerosis [31]. We speculate that the up-regulation of sialic acid and fucose residue expression in HP-PRRSV-infected PPMVECs would contribute to the occurrence of inflammation and immune response, and may result in stronger binding of infected PPMVECs with HP-PRRSV.

Finally, it should be noted that this study has investigated the surface ultrastructure and glycoprofiling of PPMVECs infected with HP-PRRSV only at 48 h post-infection. This was mainly based on our previous study [13,14], which showed that the viral titer started to increase from this time point and that most inflammatory and immune molecules were significantly enhanced at this time point. Therefore, this pilot study on surface carbohydrate expression in HP-PRRSV-infected PPMVECs was performed at 48 h post-infection. Admittedly, a single study is not enough to consolidate the results and know the kinetics of carbohydrate changes, and more studies deserve to be conducted in the future. Moreover, our main focus is on the HP-PRRSV, and the comparison between it and the conventional PRRSV was not analyzed. Considering their different virulence, we think that the surface carbohydrate changes induced by them in PPMVECs would be different. Thus, further study may contribute to uncovering the mechanism underlying their different virulence.

## 5. Conclusions

In conclusion, HP-PRRSV infection of PPMVECs significantly damages the cell surface structures, especially causing the decrease in complex N-glycans and the increase in poly-N-acetyllactosamine. Our findings suggest that changes in cell surface carbohydrate chains are an important factor causing the dysfunction of HP-PRRSV-infected PPMVECs. This study reveals the pathogenesis of HP-PRRSV from the perspective of surface carbohydrate chains of PPMVECs and provides experimental data and valuable clues for comprehensively understanding its pathogenesis.

## Figures and Tables

**Table 1:** The fluorescence intensity fold change of significantly differentially expressed lectin sites in HP-PRRSV HN and JXA1 strain-infected PPMVECs.

	PHA-L	PHA-E	DSA	LCA	SNA	UEA-I	RCA-I	LEL	STL
PRRSV HN/Mock	0.60 **	0.64 **	0.66 **	0.66 **	2.20 *	3.39 ^−^	2.17 **	1.73 **	3.97 **
PRRSV JXA1/Mock	0.83 ^−^	0.56 **	0.61 **	0.64 **	0.92 ^−^	3.66 **	4.46 **	1.88 **	5.86 **

Statistical analysis was performed by Student’s *t*-test. * *p* < 0.05; ** *p* < 0.01; “^−^” *p* > 0.05.

## Data Availability

The datasets generated for this study are available on request to the corresponding author.

## Supplementary Materials

The following supporting information can be downloaded at: https://www.mdpi.com/article/10.3390/v14112569/s1, Table S1: Lectin list in this study.
