# Supplementary material for: Effects of Highly Pathogenic Porcine Reproductive and Respiratory Syndrome Virus Infection on the Surface Glycoprofiling of Porcine Pulmonary Microvascular Endothelial Cells
Source: Viruses. 2022 Nov 20;14(11):2569. doi: 10.3390/v14112569 (PMC9695484; doi:10.3390/v14112569)
Supplement: Supplementary file 1 [file viruses-14-02569-s001.zip › viruses-2035814-supplementary.pdf]

Table S1. Lectin list in this study

NO. Name	Lectin	Full name
1	ConA	Concanavalin A
2	GNA	Galanthus nivalis Agglutinin
3	PHA-L	Phaseolus vulgaris Leuko agglutinating Phytohem Agglutinin
4	PHA-E	Phaseolus vulgaris Erythoagglutinating Phytohem Agglutinin
5	DSA	Datura stromonium Agglutinin
6	LCA	Lens culinaris Agglutinin
7	MAL-I	Maackia amurensis Leuko Agglutinin-I
8	MAL-II	Maackia amurensis Hem Agglutinin-II
9	SNA	Sambucus nigra Agglutinin
10	AAL	Aleuria aurantia Leuko Agglutinin
11	UEA-I	Ulex europeus Agglutinin-I
12	LTA	Lotus tetragonolobus Agglutinin
13	RCA-I	Ricinus communis Agglutinin-I

14	ECL	Erythrina cristagalli Agglutinin
15	PNA	Peanut Agglutinin
16	Jacalin	Jacalin
17	GSL-I	Griffonia (Bandeiraea) simplicifolia Agglutinin-I
18	WFA	Wisteria floribunda Agglutinin
19	SBA	Soybean Agglutinin
20	HPA	Helix pomatia Agglutinin
21	VVL	Vicia villosa Agglutinin
22	DBA	Dolichus biflorus Agglutinin
23	GSL-II	Griffonia (Bandeiraea) simplicifolia Agglutinin- II
24	WGA	Triticum vulgaris Wheat germ Agglutinin
25	LEL	Lycopersicon esculentum (Tomato) Agglutinin
26	STL	Solanum tuberosum Potata Agglutinin
